# Medical Opinion and Movement

**Published:** 1907-09-21

**Authors:** 


### Medical Opinion and Movement.

On the occasion of a garden party at the Ashton-under-Lyne District Infirmary, Mr. William Thorburn, surgeon to the Manchester Royal Infirmary, made some striking comparisons between the surgical work at the present time and that carried out 25 years ago in the Infirmary. Twenty-five years ago the number of operations performed by one surgeon within a certain time was 56, while now the number performed in the same time was 205. Out of the 56 cases there were 12 deaths, and of the 205 cases the deaths were still 12. Twenty-five years ago only five operations were performed on internal organs in six months, with two deaths, or a mortality of 40 per cent. To-day 73 operations were performed in the same time, with six deaths. Mr. Thorburn showed, further, the advance made in the conservative treatment of limbs. Twenty-five years ago a considerable proportion of the operations consisted in amputations. As many as 16 amputations were performed out of the comparatively small total of operations, and of these eight died, showing a mortality of 50 per cent. At the present time among the far larger number of operations there were only eight amputations and no deaths. He pointed out that the periods selected for comparison were since the introduction of anæsthetics and antiseptics, and the results were the gradual outcome of a steady and persistent study of disease.

The medical profession in Natal is considerably disturbed over a threatened increase of special taxation. For some years general practitioners have been compelled to take out an annual licence to practise in Natal, and it is now proposed to double the fee for the license, raising it to the amount of £10, and to put a further tax of £2 10s. upon all medical men who dispense their own prescriptions. A deputation from the Natal branch of the British Medical Association has recently waited upon the Government to protest against these proposals. The deputation pointed out that the annual license has always been regarded as a great injustice to the profession in view of the amount of unrequited service which the profession rendered to the public and to the State. To double such a tax would be to double the injustice and ingratitude of those who impose it. The list of gratuitous services which medical men render is no small one, and includes the services of honorary medical officers to hospitals, almshouses, benevolent societies, etc., gratuitous attendance on all indigent persons, there being no Poor-law in the Colony, and the services of professional members of the Militia Medical Corps, who train the men of the corps in their medical duties, and are prepared to quit their practices for active service. It was further shown by the deputation that the retrenchments of the Government had already seriously affected the incomes of district health officers, and at the same time they complained that the profession had not received such protection from the State as the annual tax entitled them to expect. The Government is no doubt hard pressed to find fresh sources of revenue, but an attempt to exploit the profession in this way seems altogether unfair and unjustifiable.

The abnormal growth of hair on the face is undoubtedly a source of serious trouble to women so afflicted, and many are ready to undergo any amount of inconvenience, and even pain, to be rid of the disfigurement. Up to the present electrolysis has been found the most efficacious method of treatment for hypertrichosis, but it cannot be carried out when the growth is extensive, and it is by no means a painless procedure. Moreover, however carefully it is applied it is liable to leave small scars, which are only relatively less disfiguring than the original hairs. M. Albert Weil has adopted a special method of *x*-ray treatment, and claims to have had considerable success with it. He applies the *x*-rays in accordance with the directions of MM. Sabouraud and Noire so as to determine epilation without any erythema or other effect on the skin. In the course of two or three weeks, as in the case of the treatment of ringworm, epilation is complete, but it is of course only temporary. The treatment must therefore be repeated every month for a year. After this time one or two treatments in the course of the next six months are generally sufficient to effect a complete cure, and the hairs no longer recur. Such a method of treatment must be tedious in the extreme, but the fair sex have undoubtedly a large store of patience in such matters, and the results are, according to M. Weil, most satisfactory. The utmost care is, of course, necessary in regulating the dosage of *x*-rays in order to avoid a dermatitis of the skin of the part affected.

It is generally held that a fall in blood pressure is the chief determining factor of a fainting fit. Dr. Hornung has been fortunate enough to be able to make some direct observations on the subject on two separate occasions. On the one occasion he was taking a pulse tracing when the patient fainted, and in the second instance he was recording the blood-pressure preliminary to a venesection when the same thing occurred. In both cases the instruments were in position on the arms, so that exact comparisons could be made between the records before and during the faint. The first patient was suffering from myocarditis, and had an irregular pulse with intermissions. The tracing taken during the swoon showed a marked diminution in the rise and fall of the curves, a slowing of the pulse from 72 to 66, and a complete disappearance of the intermissions. Dr. Hornung explains these changes by a fall in the blood-pressure. The second case affords more direct proof of this fall. The patient was a man with cardiac hypertrophy, arteriosclerosis, and high blood-pressure. In consequence of severe headaches, it was proposed to resort to venesection. Dr. Hornung first took a blood-pressure curve and left the instrument in position in order to observe the effect of the venesection on the blood-pressure of the patient. Alarm at the prospect of venesection caused the patient to faint. The operation was in consequence postponed, and Dr. Hornung observed instead the change of pressure caused by the fainting. There was a diminution in blood-pressure amounting to about 100 mm. of mercury.

